# Gold Nanoparticles in Diagnostics and Therapeutics for Human Cancer

**DOI:** 10.3390/ijms19071979

**Published:** 2018-07-06

**Authors:** Priyanka Singh, Santosh Pandit, V.R.S.S. Mokkapati, Abhroop Garg, Vaishnavi Ravikumar, Ivan Mijakovic

**Affiliations:** 1 The Novo Nordisk Foundation Center for Biosustainability, Technical University of Denmark, 2800 Kgs. Lyngby, Denmark; prisin@biosustain.dtu.dk (P.S.); abhgar@biosustain.dtu.dk (A.G.); vairav@biosustain.dtu.dk (V.R.); 2 Systems and Synthetic Biology Division, Department of Biology and Biological Engineering, Chalmers University of Technology, 41296 Chalmers, Sweden; pandit@chalmers.se (S.P.); ragmok@chalmers.se (V.R.S.S.M.)

**Keywords:** gold nanoparticles, cancer, protein corona, biocompatibility, photoimaging, drug delivery, photothermal therapy, photodynamic therapy, clinical trials, toxicology

## Abstract

The application of nanotechnology for the treatment of cancer is mostly based on early tumor detection and diagnosis by nanodevices capable of selective targeting and delivery of chemotherapeutic drugs to the specific tumor site. Due to the remarkable properties of gold nanoparticles, they have long been considered as a potential tool for diagnosis of various cancers and for drug delivery applications. These properties include high surface area to volume ratio, surface plasmon resonance, surface chemistry and multi-functionalization, facile synthesis, and stable nature. Moreover, the non-toxic and non-immunogenic nature of gold nanoparticles and the high permeability and retention effect provide additional benefits by enabling easy penetration and accumulation of drugs at the tumor sites. Various innovative approaches with gold nanoparticles are under development. In this review, we provide an overview of recent progress made in the application of gold nanoparticles in the treatment of cancer by tumor detection, drug delivery, imaging, photothermal and photodynamic therapy and their current limitations in terms of bioavailability and the fate of the nanoparticles.

## 1. Introduction

**Figure 1:**
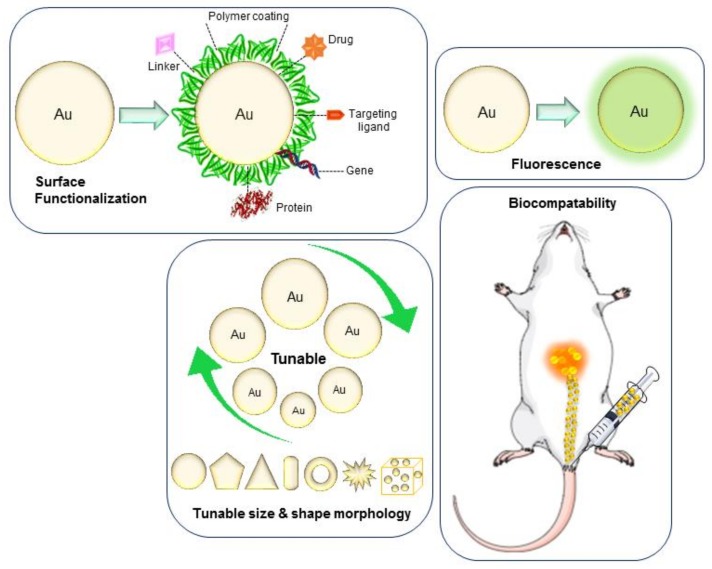
Important properties of gold nanoparticles.

With recent advances in nanotechnology and medical science, numerous nanoparticles and nanomaterials have emerged from different bulk elements such as gold, silver, iron, copper, cobalt, platinum, etc., which are synthesized either biologically or physiochemically [1,2]. The ability to manipulate nanoparticle features, such as their physical, chemical and biological properties, opens up many possibilities to explore these nanoparticles in drug delivery as image contrast agents and for diagnostic purposes [3]. Among various organic and inorganic nanoparticles, gold nanoparticles possess unique optical and Surface Plasmon Resonance (SPR) properties, due to which it has become the first choice for researchers, particularly in the biological and pharmaceutical field (Figure 1). Due to the optical properties of gold nanoparticles, they are especially utilized in ultrasensitive detection and imaging-based therapeutic techniques required for the treatment of lethal diseases, such as cancer. Cancer is a disease state caused by abnormal cell growth and is the third leading cause of mortality worldwide. According to the World Health Organization (WHO, www.who.int), cancer caused 8.8 million deaths in 2015. Current cancer treatment is based on chemotherapeutic drugs, usually involving chemo or radiation therapy, with the aim to kill the cancer cells [4,5]. However, these treatments often result in several side effects due to the damage caused to the surrounding healthy tissues. In addition, delays in diagnosis and a high incidence of relapse result in lower survival rates. Treating cancer cells by utilizing a nanoparticle-based drug delivery approach plays a key role in overcoming the limitations of conventional treatment methodologies by providing simultaneous diagnosis and treatment [4]. Consequently, a considerable amount of research focusing on gold nanoparticle-based nanocarrier development and their potential applications in cancer biology and nanomedicine has been carried out [6]. In this review, we focus on providing further new insights for exploring the gold nanoparticle applications as a tool in cancer diagnostics and treatment.

**Figure 2:**
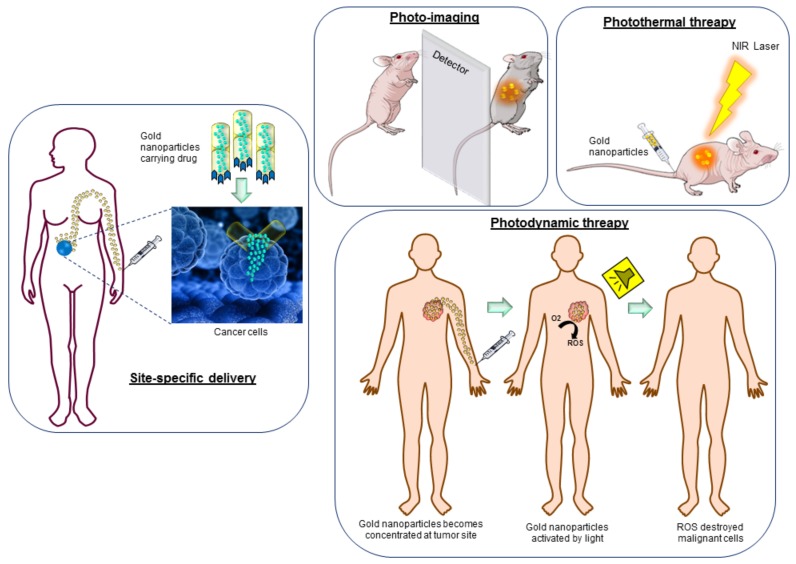
Different approaches of gold nanoparticles in cancer diagnosis and treatment.

Typically, gold nanoparticles of controlled size and shape have been synthesized by various physical (microwave and ultraviolet (UV) irradiation, laser ablation), chemical, and biological ways. Chemical synthesis generally utilizes chemicals and solvents, which are associated to environmental and human health impacts. In addition, it demands extreme conditions (e.g., pH, temperature) which are not optimal [7,8,9]. On the other hand, biological nanoparticle synthesis (plants and microorganisms mediated) is a relatively new, eco-friendly, and promising area of research with a considerable potential for expansion [10,11,12]. Numerous medicinal plants have shown potential to produce stable gold nanoparticles within a few seconds [11,13,14,15]. Microorganisms are also equally capable of adsorbing gold atoms and accumulating gold nanoparticles by secreting large amounts of enzymes, which are involved in the enzymatic reduction of gold ions [16,17]. These biologically synthesized gold nanoparticles have become an attractive and potential option to explore as a tool for biosensors, immunoassays, targeted drug delivery, photoimaging, photothermal therapy (PTT), and photodynamic therapy (PDT) (Figure 2). Interestingly, in human cancer and cell biology, various types of gold nanoparticles, such as gold nanorods, nanocages, nanostars, nanocubes, and nanospheres, have become effective tools. Their application in cancer diagnostics and therapeutic development is due to their favorable optical and physical properties that provide a potential platform for developing cancer theranostics. The optical properties of gold nanoparticles rely on SPR. In principal, SPR is a process whereby the electrons of gold resonate in response to an incoming radiation, causing them to both absorb and scatter light. In addition, some specifically shaped gold nanoparticles contribute to photon capture cross sections that are four to five-fold greater than those of photothermal dyes. These attributes are exploited to obtain localized heating either to destroy the cells or for drug release, underlying the therapeutic applications. In addition, gold nanoparticles possess tunable properties, which allow for the synthesis of nanoparticles of specific size and desired shape, resulting in a plasmonic resonance shift from 520 to 800–1200 nm (complex shapes) [18]. Susie et al. showed the change in optical properties and resonance of gold nanoparticles (ranging from 500 to 1200) by slightly changing the nanoparticles’ shape from nanospheres of 15–30 nm to nanorods of 2.5–7.5 Aspect ratio (AR.) [19] The range between 800 and 1200 is therapeutically useful because the body tissue is moderately transparent to Near Infra-Red (NIR) light, thereby providing an opportunity for therapeutic effects in deep tissues by photothermal and photoimaging approaches. Another important property is the available surface area. It is well known that the surface area of nanoparticles is inversely proportional to their size, which results in a large surface area to volume ratio. In other words, nanoparticle have a large surface area available for drug loading, conjugation, or binding of any gene or biological moiety of choice, thus increasing drug solubility, stability, and pharmacokinetic parameters [20]. The available surface area also plays a critical role for the application of gold nanoparticles in cancer diagnostics, specifically in photo-imaging and photothermal therapy. In photothermal therapy, smaller nanoparticles are preferred as light is mainly adsorbed by the nanoparticles and thus efficiently converted to heat for destruction of cell, whereas in photo-imaging, lager nanoparticles are preferred because of their higher scattering efficiency. In addition, biological responses to nanoparticles tend to scale with surface area. This means that when nanoparticles are exposed to a biological environment, such as serum or plasma, more proteins from the surroundings bind to small nanoparticles with a larger surface area-to-volume ratio than to those with a larger size and a smaller surface area-to-volume ratio. In parallel with the above-mentioned properties, the tailored surface functionalization of gold nanoparticles has also evinced considerable interest. The possibility to conjugate gold nanoparticles with a variety of biologically active moieties, especially with amine and thiol groups, provides possibilities for important biomedical applications ranging from diagnostics, targeting specific delivery of drugs/genes, imaging, and sensing for electron microscopy markers [21,22].

**Figure 3:**
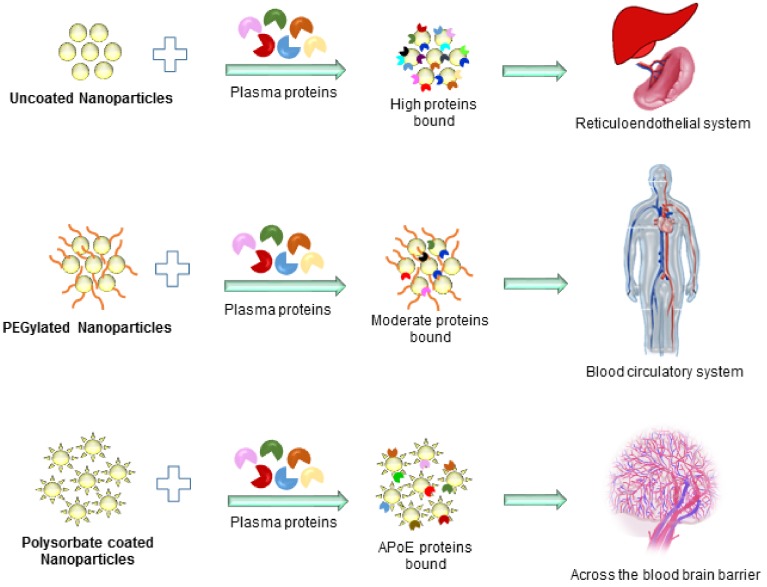
Distribution of nanoparticles with varying coatings and bound proteins. PEG = poly-ethyleneglycol.

**Figure 4:**
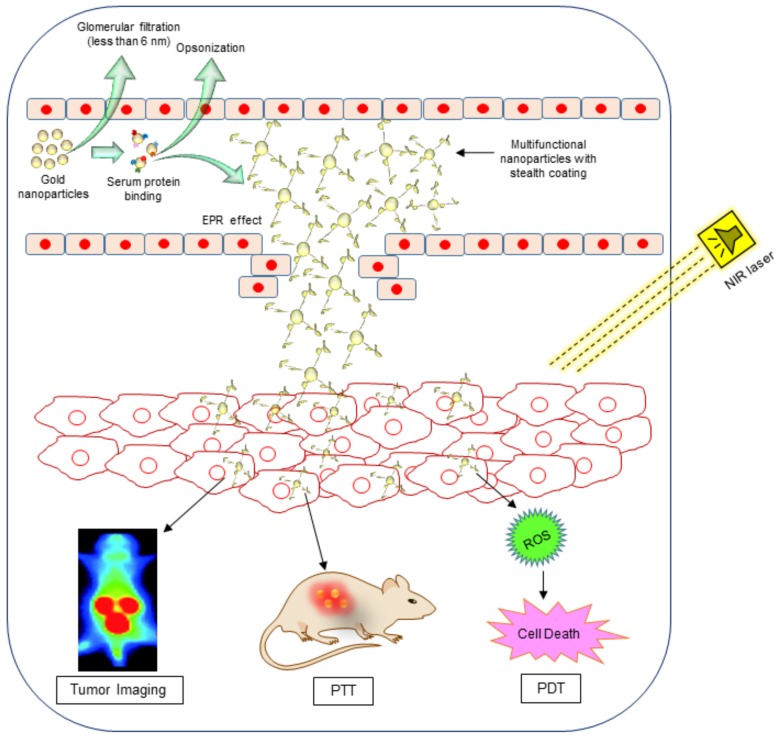
Systemic delivery of multifunctional gold nanoparticles for cancer bioimaging, photothermal therapy (PPT), and photodynamic therapy (PDT). EPR = enhanced permeation and retention; NIR = near infra-red; ROS = reactive oxygen species.

Despite all these benefits, biocompatibility of gold nanoparticles is a crucial factor to take into account prior to clinical applications. Although the inert nature of gold nanoparticles makes them relatively biocompatible, the cytotoxicity of the nanoparticles, which is more or less dependent on their shape, size, surface properties, and chemical composition, has to be further evaluated. Once the particles are internalized by the cells, the proteins present in the physiological environment form a coating called a “corona” on the surface of the nanoparticles, resulting in a nanoparticle-protein complex [23]. This protein corona is quite complex and variable in structure and plays a key role in the biodistribution of nanoparticles throughout the body. Dobrovolskaia et al. reported that untreated plasma usually contains approx. 3700 proteins, and the gold nanoparticles that come in contact with the plasma form a “protein corona complex” containing fewer than 100 proteins [24]. Gold nanoparticles inside the corona complex contain opsonins on their surface, which are recognized by the immune cells (part of reticuloendothelial system (RES)). These proteins ultimately determine the route of nanoparticle internalization and eventually affect the fate of the nanoparticles in the body (i.e., rate and route of clearance from the bloodstream and body, volume of distribution, organ disposition, etc.) (Figure 3) [25]. So far, various proteins have been reported to have been isolated from the corona complex in the plasma, such as albumin, fibrinogen, Immunoglobulin G (IgG), Immunoglobulin M (IgM), transferrin, etc. This corona complex can cause changes in particle size and charge, which in turn affect the internalization process into the macrophages of the RES and the overall distribution in the body. Certain proteins allow macrophages to easily recognize nanoparticles; for example, IgG opsonins, and fibrinogens are reported to promote phagocytosis and nanoparticles removal from the body [26], whereas dysopsonins such as albumins are reported to cause prolonged blood circulation of nanoparticles [27]. To prevent the nanoparticles from immune recognition, scientists introduced a process called “PEGylation”. In this approach, nanoparticles “hide” by masking their surface with a poly-ethyleneglycol (PEG) layer. This saves them from immune recognition, in essence prolonging their blood circulation. PEGylation can be done by covalent linking that entraps or adsorbs PEG chains onto the surface of the nanoparticle. Once the nanoparticles are internalized, they can be used in tumor imaging, PTT, and PDT. Although PEGylation can help in avoiding rapid recognition by the RES, complete avoidance is rarely achieved as nanoparticles may still be recognized and taken up by the RES system (Figure 4). Though a lot of work in this regard has been conducted, many challenges remain and must be tackled before PEGylation can be put into practice. In this review, we summarize the importance and advantages of gold nanoparticles and their utilization in several aspects of cancer therapeutics.

## 2. Gold Nanoparticles as Drug Carriers

Traditional drug delivery approaches for chemotherapeutic drugs, i.e., oral or intravenous administration, result in the dissemination of the drug in the whole body, with only a fraction of the drug reaching the tumor site. This, however, can have side effects on healthy tissues and organs. This problem of side effects is circumvented by targeted drug delivery approaches, which can be defined as a process in which a specific biologically active compound or drug is released at a specific location in a controlled manner. The development of nanoparticles has opened up enormous possibilities for drug delivery. Due to their small size, they can efficiently pass through the capillaries to reach their target cells. The chemotherapeutic drugs can be loaded or attached to nanoparticles and can be targeted either passively or actively to the tumor site. Tumor tissue generally has a leaky vasculature, which allows the nanoparticles to accumulate easily. This is also known as the enhanced permeation and retention (EPR) effect. This form of passive targeting utilizes the pathophysiological properties of the tumor tissue. However, there are certain limitations to this approach, which include arbitrary targeting and inefficient dispersion of drugs in tumor cells. Additionally, not all tumors exhibit the EPR effect. In active targeting, the ligands of tumor specific biomarkers such as monoclonal antibodies, aptamers, peptides, and vitamins are conjugated on to the nanoparticle surface. These ligands then interact with their receptors on the tumor cells, allowing for endocytosis and subsequent release of the drug. Thus, active targeting offers a higher probability of endocytosis as compared to the passive targeting approach [28].

Gold nanoparticles have caught the attention of scientists for their use as drug carriers because of their SPR, optical, and tunable properties. They can be prepared in a broad range of core sizes (1 to 150 nm), which makes it easier to control their dispersion. The presence of a negative charge on the surface of gold nanoparticles makes them easily modifiable. This means that they can be functionalized easily by the addition of various biomolecules such as drugs, targeting ligands, and genes. In addition, the biocompatibility and non-toxic nature of gold nanoparticles makes them an excellent candidate for their use as drug carriers [29,30]. For example, methotrexate (MTX), which has been used to treat cancer for decades, upon conjugation with gold nanoparticles displayed higher cytotoxicity towards numerous tumor cell lines as compared to that of free MTX. MTX was observed to accumulate in the tumor cells at a faster rate and to a higher level when conjugated with gold nanoparticles [31]. Another drug, doxorubicin (DOX), when bound to gold nanoparticles via an acid labile linker, showed enhanced toxicity against the multi drug resistant MCF-7/ADR breast cancer cell line, thus overcoming the multi drug resistance to some extent due to the enhanced uptake of the gold nanoparticle-tethered drug followed by its responsive release within the cell [32]. In the past, peptide-drug-conjugates (PDCs) have been investigated for their use as anticancer agents [33,34,35,36]. However, their stability in the blood, liver, and kidneys pose a significant challenge to their successful use as an anticancer molecule. Recently, it was shown that this difficulty can be by-passed by conjugating these PDCs to gold nanoparticles. The authors reported an increase in the half-life of PDCs from 10.6–15.4 min (administered alone), to 21.0–22.3 h (upon conjugation with gold nanoparticle), while retaining cytotoxicity [37]. Apart from synthetic drugs, phytochemicals have also shown the potential of being used as anticancer drugs but, similar to PDCs, they too have certain problems such as low specificity, short half-life, fast clearance rate, and inefficient cell penetration. These problems in using phytochemicals can be by-passed by conjugating them to gold nanoparticles. For example, kaempferol (a phytochemical) conjugated to gold nanoparticles displayed both significantly higher apoptosis and inhibition of angiogenesis in MCF-7 breast cancer cells as compared to kaempferol alone [38]. Table 1 lists some of the studies performed to investigate the anti-tumor applications of gold nanoparticles in drug delivery.

## 3. Gold Nanoparticles in Photothermal Therapy and Photoimaging

In this section, we discuss the photothermal and photoimaging applications of gold nanoparticles, which are still under development. Photothermal therapy (PTT) is referred to as photon mediated induction of the localized therapeutic temperature that can stimulate hyperthermic physiological responses. In simple terms, it melts the tumor in molten gold. This therapy uses metal nanoparticles (such as gold) that can exhibit SPR and efficiently convert light into heat [48]. The extent of heat generation is directly proportional to the incident excitation power and the nanoparticle itself. In addition, the wavelength of plasmonic absorption can be tuned by optimizing the surface properties, shape, and size of the nanoparticles. The nanoparticles used in PTT are generally rod-shaped or shell-shaped gold nanoparticles, and when they are being introduced in to a biological environment, like any other foreign material, the cellular uptake can be limited. Several groups have reported different methods of tagging, functionalization, etc. based on which method seems to largely improve the uptake [49]. Near infra-red (NIR) light is chosen for PTT due to minimal absorption by the tissues at these wavelengths (650–900 nm) [50], which is enough to induce cytotoxic damage [51]. There have also been other studies on PTT where gold nanoparticles were used at a maximum absorption of 795 nm and also on branched gold nanoparticles functionalized with nanobodies that can effectively kill cancer cells leaving healthy cells unharmed [52,53]. Lin et al. carried out the first demonstration of gold nanoparticles in PTT where 30 nm gold nanospheres conjugated with IgG antibodies were used [54]. This in vitro demonstration was specifically targeted towards CD8 (cluster of differentiation 8) receptors on lymphocytes where 95% of the gold nanoparticle (AuNP)-IgG treated cells were killed. The most successful gold nanoparticles that are approved by the Food and Drug Administration (FDA) and are in ongoing human pilot studies are PEGylated gold nanoparticles. They have shown enhanced accumulation and absorption at the tumor site in the NIR region [55]. According to the reported studies, more than 50% of the mice treated with a single administration of PEGylated gold nanoparticles and a single 10 min laser exposure showed no recurrence of the disease even after 2 weeks [55]. Further optimized PEGylated silica gold nanocore shells are being studied for the head and neck tumors in humans (Nanospectra Biosciences INC NCT00848042).

Photodynamic therapy (PDT) is another form of cancer treatment that utilizes light, photosensitizers and oxygen from the tissues. Unlike PTT, which is oxygen independent, PDT is completely dependent on the availability of tissue oxygen. In the case of PDT, a photosensitizing agent such as porphyrin is intravenously injected into the tissues and excited by specific wavelengths, leading to the energy transfer that generates reactive oxygen species (ROS) and causes cell death by apoptosis. PDT typically has a very low chance of causing mutations as the ROS do not accumulate in the cell nuclei. Recently, PTT and PDT were applied simultaneously in melanoma-xenografted mice. The lipid-coated gold nanocages were exposed to a 980 nm continuous wave (CW) laser, which raised the temperature (~10 °C) and generated the singlet oxygen, ultimately damaging the tumor growth [56]. The biggest drawback with PDT is that the photosensitizers are not generally soluble in the physiological environment, which inhibits their uptake by the diseased tissues. Nevertheless, some studies do show that these photosensitizers can be conjugated to the gold nanoparticles, thereby facilitating their uptake and delivery to the cancer tissues [57]. On the other hand, PDT has its own advantages in terms of minimal invasiveness, no cumulative toxicity, and reduced morbidity [58], which has proven effective for lung cancer [59], head and neck cancers [60], and skin cancer [61]. The important and interesting aspect is that PDT and PTT can also be combined to have an effective dual therapy where gold nanoparticles (nanorods and nanocages) were used in conjugation [61,62,63]. One good example is the work by Seo et al., where methylene blue loaded mesoporous silica coated gold nanorods were used for PTT/PDT dual therapy [64]. The photosensitizer was physically adsorbed on the pores of the silica shell. Upon irradiation with NIR (near infra-red) light at 780 nm, the viability of CT-26 cells (Murine colon carcinoma) was found to decrease by 31% for the cells transfected with gold nanorods, while the methylene blue (MB) loaded nanocomposites showed an 11% drop, which indicates a synergistic effect of dual therapy [64]. An important aspect that has to be noted here is the effect of PTT therapy on PDT or vice versa. Liu et al. presented a detailed analysis of this dual therapy where they clearly showed that the hypoxic environment induced by PDT does not affect PTT as PTT is an oxygen-independent therapy.

Photoimaging is an advanced technique that can help detect early stage tumors and guide the surgeons for precision treatment. One of the biggest challenges today for the surgeons is to have a clear picture of where the tumor ends and the healthy tissue begins. During an operation, the surgeons face a nearly impossible task of deciding to what extent the tumor has to be removed: being too conservative can leave some tumor cells behind, and being too liberal can result in removing healthy tissues that can be vital. Being too conservative is the reason that most of the tumors recur with time. Magnetic Resonance Imaging (MRIs) and Computed Tomography (CT) scans are limited and can only detect tumors above a size of several millimeters or approximately 10 million cells, meaning that the tumors are detected only when they reach a certain threshold. Photoimaging is a novel approach in cancer treatment where millions of functionalized gold nanoparticles are site specifically injected into the tumor, where they specifically bind to the cancer cells and scatter (shine), making it easier for the surgeons to identify the tumor and healthy cells. Gold nanoparticles (nanorods, nanocages, and nanoshells) are known to be the best available photo imaging nanoparticles for cancer therapeutics due to their bio inertness and their ability to provide increased spacial and temporal resolution for imaging [65].

## 4. Recent Advance to Explore Gold Nanoparticles in Clinical Trials

Very few clinical trials are being actively carried out for the approval of gold nanoparticles for cancer diagnostics and therapy (Table 2) [66,67]. According to the literature, the FDA has approved few gold nanoparticle-based technologies for diagnostic and therapeutic purposes in medicine [55,68]. The cytotoxicity of gold nanoparticles is highly dependent on the size and morphology of the particles, environmental scenario, and the method of production [66,69,70]. One of the clinical trials being carried out by Astra Zeneca in partnership with Cytimmune mainly focuses on gold nanoparticle-based cancer treatment (http://cytimmune.com/#pipeline). Their first phase of trials was successfully completed. Aurimune (CYT-6091) was used as a vehicle to deliver the recombinant human tumor necrosis factor alpha (rhTNF) into tumors, which disrupted the blood vessels, enabling chemotherapeutic drugs to penetrate the tumor and damage the cancer cells. Safe delivery of highly effective doses of rhTNF to tumor cells was observed [71]. The authors also found that the dose of rhTNF administered after immobilization to gold nanoparticles could be three times higher than its usual dose without any toxic effect [71]. The PEG layer also decreased the uptake of nanoparticles by the mononuclear phagocytic system (MPS) and aided in their accumulation in the tumor masses via the EPR effect.

Due to the favorable ability of gold nanoparticles to absorb NIR-light, interest towards PTT has increased of late. Researchers are mainly focusing on the photothermal conversion efficiencies, selective targeting of cancer cells, enhanced cancer cell destruction, and in vivo bio-distribution of the nanoparticles [72]. For example, Aurolase^®^, developed by Nanospectra, are silica-gold nanoshells coated with (poly)ethylene glycol (PEG) and designed to thermally ablate the solid tumors following stimulation with a NIR light source. The absorption of light leads to an increase in the local temperature, which thermally dissolves the solid tumors [73]. In a recent clinical trial, AuroLase^®^ particles were used in a localized therapy for the treatment of primary or metastatic lung tumors (NCT01679470). Another trial, last updated in September 2017 (NCT00848042), was reported as being completed for the treatment of patients with refractory and/or recurrent tumors for head and neck cancer. A currently active trial uses AuroLase^®^ as an imaging technology during focal ablation of prostate tissue using nanoparticle-directed laser irradiation. Since no active drug is used in this approach, AuroLase^®^ is activated externally at the target site, thus avoiding any toxicity towards the normal cells. This is the only ultra-focal tumor ablation therapy designed and dedicated to maximize the treatment efficacy while causing minimum side effects. In addition to this, several gold nanoparticle-based theranostics have recently advanced to clinical trials. One such currently ongoing trial is aimed at the safety evaluation of NU-0129, a spherical nucleic acid (SNA) formulation composed of small interfering RNAs (siRNAs) targeting the Bcl-2-like protein 12 (BCL2L12) sequence and conjugated to gold nanoparticles, that has potential antineoplastic activity (NCT03020017). Kharlamov et al. studied the safety and feasibility of two delivery techniques for gold nanoparticles for the treatment of atherosclerosis. The first is a PTT approach with silica-gold nanoparticles and the second is a magnetic navigation approach with silica-gold iron bearing nanoparticles (NCT01270139) [74]. Results obtained in this trial suggested that PTT using gold-silica nanoparticles is associated with a significant regression of coronary atherosclerosis and an acceptable level of safety for clinical practice. CNM-Au8 is a gold nanocrystal suspension drug developed by Clene Nanomedicine (Salt Lake City, UT, USA) for the demyelinating disorder neuromyelitis optica (NMO) (NCT02755870). A randomized placebo controlled trial is ongoing in healthy individuals to evaluate the safety, tolerability, and pharmacokinetics of CNM-Au8. Another gold-nanoparticle based clinical trial is currently underway, with the aim to evaluate the feasibility of a novel method in oncology involving breath analysis with a nanosensor array for identifying gastric diseases (NCT01420588). It has been suggested that the nanosensor array could provide the missing non-invasive screening tool to distinguish gastric cancer and related precancerous lesions [75]. A clinical trial on another novel diagnostic approach (electronic nose sensor) with gold nanoparticles is ongoing for the evaluation of its performance in the diagnosis of pulmonary arterial hypertension (NCT02782026).

## 5. Current Limitations

As described above, gold nanoparticles do show promise and potential to be used in cancer diagnostics and therapeutics. Nevertheless, it is imperative to consider the other side of the coin, i.e., unintended side effects on human health. A number of individual studies previously addressed the cytotoxicity, effect of size on toxicity, efficacy, biodistribution, retention time, and physiological response of nanoparticles. However, many of them are seen to contradict one another. Absence of coherent information on the actual effect of nanoparticles could have delirious effects and a negative impact on human health. While the discussed issues in general are applicable to any nanoparticle, examples described below are specific to gold nanoparticles.

Toxicity: The toxicity of gold nanoparticles to biological systems has always been an issue of concern. Properties of gold nanoparticles such as shape, size, surface chemistry, targeting ligand, elasticity, and composition largely influence their toxicity. This, in combination with the complexity and the heterogeneity that exists amongst human cells and tissues, makes it challenging to comprehensively probe the effect and response of the biological system to the administration of gold nanoparticles. Surface charge has been reported to influence toxicity of gold nanoparticles, wherein positively charged particles were found to be more toxic than negative or neutral particles [76]. On the other hand, other groups found no toxicity induced by positively charged gold nanoparticles [77] and no toxicity of negatively charged particles [78]. This discrepancy arises due to the unique physiochemical nature of nanoparticles, and no single standardized assay is currently available that could universally be applied to test the toxicity effect of all nanoparticles. The lack of such robust standardized assays leads to varying interpretations or assumptions that limit nanoparticle administration. Toxicity assay using *Caenorhabditis elegans* (ISO 10872 method) is widely used to assess the effect of nanoparticles on multicellular organisms. Hanna et al. recently reported an artifact caused while testing the toxicity of positively charged gold nanoparticles using the *C. elegans* assay [79]. The authors initially observed growth inhibition of the nematodes when fed with *E. coli* along with a suspension of positively charged gold nanoparticles. However, they deduced that this observation was a false positive as gold nanoparticles heteroagglomerate with *E. coli* cells, influencing the ability of the nematodes to feed. On repeating the assay in the absence of *E. coli*, the authors observed a reduced toxicity effect, illustrating unforeseen artifacts that could occur in such widely used toxicity assays. Ginzburg et al. showed a synergistic toxicity effect induced by gold nanoparticles in the presence of additives such as surfactants, while the individual components separately exhibited low toxicity [80], underlining the importance of identifying strategies for selecting safe nanoparticles-additive/ligand/modifier combinations. Gold nanoparticles were also shown to have species-specific differences with respect to biodistribution, pathophysiologic response, and retention time. Bahamonde et al. observed that gold nanoparticle-treated mice and rats responded differently, wherein a number of rats died on gold nanoparticle administration while no fatality was seen amongst the mice [81]. Additionally, the authors also noticed a relatively higher accumulation of the gold nanoparticle in rats as compared to the mice, highlighting the fact of differential physiological response even amongst closely related groups.

Size and Biodistribution: Apart from toxicity assessment, size and biodistribution of nanoparticles are also significant factors to take into consideration. Tang et al. reported increased cytotoxicity of smaller gold nanoparticles (8 nm) coated with reduced glutathione when tested on a human hepatic cell line as compared to that of the larger particles (37 nm) [82]. On the other hand, Rosli et al. recorded that 50 nm gold nanoparticles exhibited higher cytotoxicity in a breast cancer cell line as compared to their 13 and 70 nm counterparts [83]. Connor et al. studied the cytotoxicity of a series of gold nanoparticle sizes ranging from 4 to 18 nm on human leukemia cells and found that none of the sizes were not harmful to cellular function [84]. Liang et al. showed that PEG-coated gold nanoparticles of 4.8 nm had the highest toxicity effect on Hela cells whereas the 12.1 and 27.3 nm counterparts showed low toxicity and the 46.6 nm counterpart exhibited absolutely no toxicity [85]. Li et al., however, reported that regardless of the size of the nanoparticles, the observed cytotoxicity was due to dose-dependency [86]. Sonavane et al. noticed a similar accumulation of gold nanoparticles in the liver while testing the effect of nanoparticle size on biodistribution. The authors observed that gold nanoparticles of all sizes mainly accumulated in organs like liver, lung, and spleen. The 15 nm particles accumulated in tissues including blood, liver, lung, spleen, kidney, brain, heart, and stomach whereas much larger particles (200 nm) showed a very minute presence in organs including blood, brain, stomach, and pancreas [87]. With respect to biodistribution, Fraga et al. assessed the biodistribution of ~20 nm citrate- and pentapeptide CALNN (cysteine–alanine–leucine–asparagine–asparagine) -coated gold nanoparticles and found them to mainly accumulate in liver [88]. Cho et al. analyzed gold contents of 13 nm PEG-coated gold nanoparticles and found them to accumulate in the liver and spleen [89]. Li et al. showed the accumulation of larger PEG-coated gold nanoparticles (42.5 and 61.2 nm) in the liver. Additionally, the authors also observed a longer retention time (poor elimination rate) possibly causing further safety issues [86]. Aside from those issues mentioned above, it is vital to take into account the non-biodegradability and non-porous properties of gold nanoparticles which would likely have an effect on the pharmacokinetics [90].

Thus, currently, there is controversy and inconsistency regarding the potential of gold nanoparticles for clinical applications, and there is an inherent need for the development of universally applicable methods to evaluate the biocompatibility of gold nanoparticles and to have a firm understanding of their interaction with the living system. Lastly, it is also worthwhile to reflect on whether the cost of synthesis would justify the underlying therapeutic capabilities of gold nanoparticles (concept discussed in general in [91]).

## 6. Concluding Remarks and Future Perspectives

In this review, we highlighted the recent advances in the development and application of gold nanoparticles in cancer diagnostics and treatment. Gold nanoparticles’ optical properties, easy synthesis, possible control over size and shape, colloidal stability, and the ability to tune the surface chemistry to achieve easy conjugation with biological moieties make them favorable for biomedical applications. Importantly, surface and core properties of gold nanoparticles can be optimized for individual and multifold applications including molecular recognition, chemical sensing. and imaging. Thus, photoimaging and PTT are attractive approaches to treat the tumor cells. Millions of functionalized gold nanoparticles can be released into the blood stream and bind to specific cancer cells, and in turn either aid in treatment via PTT or enable photoimaging for the successful removal of the tumor by operation. Despite these advances with gold nanoparticles, there is still a requirement for more cost-effective gold nanoparticle-based systems, which will allow cancer diagnosis and treatment at an early stage with a high level of specificity. The very first step for developing such a nanosystem is to understand the nanoparticle properties described above and the nature of the protein corona complex, which eventually influences its uptake and distribution throughout the body. Gold nanoparticles are actively employed in drug delivery, PTT, and in imaging techniques, despite these approaches having limitations owing to non-specific binding and the potential activation of the normal host immune response. These issues have been tackled by using PEGylation Mask technology thereby rendering the nanoparticle surfaces inert with respect to protein absorption, thus reducing the probability of an immune response. Paradoxically, if a particle is so well coated that it becomes “invisible” to the immune system, it will also probably lose its ability to bind to specific receptors. To overcome these in vivo barriers, gold nanocomplexes are further modified with targeting ligands and bioresponsive linkers. However, extensive modification may cause unwanted toxic effects. In principle, active targeting of the nanoparticle is also possible, although further work on methods to evade the immune system en route to the target is a prerequisite. Thus, there are a number of critical issues that need to be addressed such as reproducible and reliable manufacturing methods/assays, long-term health effects, stability, and cellular and immune responses. This calls for continued research in the development of the techniques described above, particularly with respect to active targeting and PTT. Moreover, nanoparticle fate is an equally important and substantial aspect to acknowledge before exploiting it in the clinical trial applications. Hence, in this respect, a great deal of research will be required to focus on internalization of the nanoparticles, their subsequent localization, relevant immunological response, and, most importantly, their excretion from the human body.

## Figures and Tables

**Table 1:** Anti-tumor applications of gold nanoparticles in drug delivery.

Nanoparticle	Nanoparticle Size (nm)	Outcome	Cell Lines	Ref.
MTX-AuNP	8–80	Higher cytotoxicity towards numerous cell lines as compared to free MTX. Suppression of tumor growth with MTX-AuNP but not with free MTX.	Lewis lung carcinoma (LL2) cells	[31]
DOX-Hyd@AuNP	30	Enhanced toxicity against multi drug resistant cancer cells.	MCF-7/ADR cancer cells	[32]
(Pt(R,R-dach))-AuNP	26.7	Platinum-tethering exhibited higher cytotoxicity as compared to free oxaliplatin that could enter the nucleus.	A549 lung epithelial cancer cell line, HCT116, HCT15, HT29, and RKO colon cancer cell lines	[39]
Tfpep-AuNP conjugated with photodynamic pro-drug Pc 4	5.1	Cellular uptake of targeted particles was significantly higher than that of the non-targeted ones.	LN229 and U87 human glioma cancer lines	[40]
CPP-DOX-AuNP	25	Higher cell death as compared to previously tested 41 nm AuNP.	HeLa cells and A549 cells	[41]
FA-Au-SMCC-DOX		Enhanced drug accumulation and retention as compared to free DOX in multi drug resistant cancer cells.	HepG2-R, C0045C, and HDF	[42]
FA-BHC-AuNP	20–60	Increased efficacy of BHC against cancer cells.	Vero and HeLa	[43]
Au-P(LA-DOX)-b-PEG-OH/FA NP	34	Enhanced cellular uptake and cytotoxicity against cancer cells.	4T1 mouse mammary carcinoma cell line	[44]
DOX@PVP-AuNP	12	Induction of early and late apoptosis in lung cancer cells and upregulation of tumor suppression genes.	A549, H460, and H520 human lung cancer cells	[45]
DOX-BLM-PEG-AuNP	10	Enhanced half-maximal effective drug concentration, providing rationale for chemotherapy using two drugs.	HeLa cells	[46]
EpCam-RPAuN	48	The biomimetic nanoparticle loaded with PTX was used in combination treatment (PTT and chemotherapy).	4T1 mouse mammary carcinoma cell line	[47]

AuNP: Gold nanoparticle, AuN: Gold nanocage, BHC: Berberine hydrochloride, BLM: Bleomycin, CPP: Cell penetrating peptides, DOX: Doxorubicin, EpCam: epithelial cell adhesion molecule, FA: Folic acid, Hyd: Hydrazone, MTX: Methotrexate, PEG: Poly ethylene glycol, PLA: Poly l-aspartate, (Pt(R,R-dach)): Active ingredient of oxaliplatin, PTT: Photothermal therapy, PTX: Paclitaxel, PVP: Polyvinylpyrrolidone, SMCC: Succinimidyl 4-(*N*-maleimidomethyl) cyclohexane-1-carboxylate, Tfpep: Transferrin peptide.

**Table 2:** Lists of clinical trials of gold nanoparticles.

Name	Materials	Application	Clinical trials.gov Identifier
AuroLase^®^	Silica-gold nanoshells coated with PEG	Laser responsive thermal ablation of solid tumors: head/neck cancer, primary and/or metastatic lung tumors	NCT00848042, NCT01679470
AuroLase^®^	Silica-gold nanoshells coated with PEG	Prostate, head and neck, lung MRI/US fusion imaging and biopsy in combination with nanoparticle-directed focal therapy for ablation of prostate tissue	NCT02680535
NU-0129	A Spherical Nucleic Acid (SNA) Gold Nanoparticle	Targeting BCL2L12 in recurrent glioblastoma multiforme or gliosarcoma patients	NCT03020017
Silica-Gold Nanoparticles	Silica-Gold Nanoparticles	Plasmonic photothermal therapy of flow-limiting atherosclerotic lesions	NCT01270139
CNM-Au8	gold nanocrystal	Evaluation of safety, tolerability, and pharmacokinetics of CNM-Au8 in healthy male and female volunteers	NCT02755870
Gold Nanoparticles	Gold nanoparticles	Sensors functionalized with gold nanoparticles Organic functionalized gold nanoparticles Detection of gastric lesions	NCT01420588
Gold Nanoparticles	Gold nanoparticles	Exhaled breath olfactory signature of pulmonary arterial hypertension	NCT02782026
